# Effect of stigma maydis polysaccharide on the gut microbiota and transcriptome of VPA induced autism model rats

**DOI:** 10.3389/fmicb.2022.1009502

**Published:** 2022-11-04

**Authors:** Xiaolei Yang, Jiyuan Li, Yang Zhou, Ning Zhang, Jicheng Liu

**Affiliations:** ^1^Department of Preventive Medicine, School of Public Health, Qiqihar Medical University, Qiqihar, China; ^2^Department of Anorectal Surgery, The Second Affiliated Hospital of Qiqihar Medical University, Qiqihar, China; ^3^College of Pathology, Qiqihar Medical University, Qiqihar, China; ^4^Research Institute of Medical and Pharmacy, Qiqihar Medical University, Qiqihar, China

**Keywords:** autism, gut microbiota, RNA-seq, 16S rRNA, stigma maydis polysaccharide

## Abstract

Stigma maydis polysaccharide (SMPS) is a plant polysaccharide that participates in immune regulation and gastrointestinal motility. Autism spectrum disorder (ASD) refers to a group of neurodevelopmental disorders, and ASD patients often present intestinal microflora imbalance problems; however, there is no effective treatment method. This study explores the effect of SMPS intervention on the gut microbiota in autism model rats as well as the potential action pathways. Female Wistar rats were intraperitoneally injected with sodium valproic acid (VPA) or normal saline at embryonic day 12.5 to establish an autism model or normal control in their offspring. The offspring prenatally exposed to VPA were randomly assigned to the VPA and the SMPS groups. The SMPS group was administered SMPS from E0.5 to postnatal day (PND) 21. We performed 16S rRNA and transcriptomics analyses to reveal the gut microbiota (GM) and differentially expressed genes in the autism model rats in response to SMPS intervention. SMPS intervention significantly improved the diversity and structure of the GM in autism model rats compared with the VPA rats. Moreover, the relative abundance of *Prevotellaceae* and *Lachnospiraceae_NK4A136_group* was increased after SMPS intervention. Transcriptome sequencing showed that 496 differentially expressed genes (DEGs) were identified after SMPS administration compared with the VPA group. Meanwhile, gene ontology (GO) enrichment analysis of DEGs was showed that the SMPS group had significant 653 GO terms. SMPS intervention had a major influence on oxidative phosphorylation, retrograde endocannabinoid signaling, thermogenesis, ribosome, protein digestion and absorption, renin-angiotensin system, calcium signaling pathway, glycosphingolipid biosynthesis-ganglio series, and propanoate metabolism pathways. Overall, this study suggests that SMPS interventions in early life may have an impact on gut microbiota, and then affect the transcriptomics levels of the hippocampal tissue in the VPA-induced autism model rats. It provides scientific evidence for the role of the microbe-gut-brain axis in ASD research.

## Introduction

Autism spectrum disorder (ASD) is a group of severe neurodevelopmental disorders that present social impairments and repetitive behavior problems. Currently, the prevalence of the disease has reached 1/59; however, its etiology remains unclear, with few therapeutic breakthroughs observed (Saurman et al., 2020). While numerous medical comorbidities have been associated with ASD, gastrointestinal (GI) problems seriously affect the normal life and rehabilitation treatment of ASD individuals (Kang et al., 2017). A strong association is observed between GI symptoms and intestinal microbiota misbalance in ASD patients (Fu and Lee, 2021). The changes in bacterial diversity and community structure of the intestinal microbiome may affect the gut-brain neural network, gut immune system and neurotransmitters and then interfere with the function of the gut-brain axis, subsequently aggravating the behavioral disorders of ASD children (Wang and Wang, 2016; Ma et al., 2019; Sgritta et al., 2019). Insights into the mechanisms of microbiome-gut-brain communication may clarify the underlying pathophysiology of ASD and lead to the discovery of novel therapeutic targets.

Constipation, abdominal pain and diarrhea are common gastrointestinal problems in children with ASD that not only worsen the autistic behavioral symptoms but also cause stress and anxiety problems (Kang et al., 2017; Ferguson et al., 2019; Madra et al., 2020, 2021). These GI symptoms of ASD children may be associated with an imbalance of gut microbiota (GM) and poor intestinal motility (Gabriele et al., 2016; Pulikkan et al., 2019). Therefore, microbially mediated therapies, specifically probiotics and fecal microbiota transplantation, have shown promise in the treatment of GI symptoms in ASD children and potential benefits to the core behavioral symptoms of autism as well (Martínez-González and Andreo-Martínez, 2020; Tan et al., 2021).

Stigma maydis polysaccharide (SMPS) is a plant polysaccharide that participates in immune regulation and gastrointestinal motility. Intestinal flora represent an important bridge in the interaction between plant polysaccharides and the human body. Polysaccharides are digested into short-chain fatty acids by intestinal flora to aid absorption and utilization, produce active metabolites with pharmacological effects, and contribute to physiological function control (Sun et al., 2020; Chen et al., 2021; Zhou et al., 2021). Meanwhile, the abundance, species, and proportion of the host intestinal microflora can all be influenced by polysaccharides. Numerous studies based on the “gut-brain axis” theory have found that the occurrence of many neurological diseases is closely associated with intestinal microbiosis (Saurman et al., 2020; Socała et al., 2021). Interestingly, polysaccharides can modulate the synthesis of neurotransmitters (such as serotonin and dopamine) in the human gut and affect their involvement in the structure and function of the nervous system by boosting the growth of helpful intestinal bacteria while suppressing the growth of harmful bacteria (Settanni et al., 2021; Sun et al., 2021; Zhang et al., 2022). Recent human genetics revealed that bear long polysaccharides were associated with autism spectrum disorder and played an important role in synapse formation, neural plasticity (Kamimura and Maeda, 2021). One study reported that clostridium bolteae levels were overabundant in intestinal tract of ASD children suffering from gastric intestinal ailments. However, it could produce a conserved specific capsular polysaccharide which might be as a vaccine to reduce or prevent clostridium bolteae colonization of the intestinal tract in autistic patients, and hopefully defend the augmentation of regressive-autism related symptoms (Pequegnat et al., 2013). Additionally, Endreffy et al. found that total glycosaminoglycans (a family of linear, sulfated polysaccharides that are associated with central nervous system development, maintenance and disorders) were significantly higher in the urine of ASD children compared to healthy controls (Endreffy and Bjørklund, 2016). However, adequate evidence is not available to explain the correlation between polysaccharides and autism, especially if polysaccharides can influence the gut-brain axis by regulating the gut microbiota. In our previous study, we found that SMPS intervention could meliorate behavioral impairments and alleviate some inflammatory responses in the gut of VPA induced autism model rats. Based on the above, this study was aimed to clarify the effect and mechanism of SMPS on the gut microbiota of autistic rats by 16S rRNA sequencing and RNA-seq technologies.

## Materials and methods

### VPA-induced autism rat model construction and SMPS intervention

SPF-grade healthy adult Wistar rats, including 12 females and 12 males, each weighing 250–280 g, were provided by the Animal Laboratory Center of Qiqihar Medical University. The rearing environment was maintained at 22 ± 2°C, humidity 50 ± 10%, natural circadian variable light, and national standard rat growth and breeding feed. All experiments were approved by the Ethics Committee of Qiqihar Medical University and operated in strict accordance with its relevant regulations (QMU-AECC-2021-62).

The establishment of the VPA-induced autism model was performed according to method of Schneider (Schneider and Przewłocki, 2005). After a week of adaptive rearing of Wistar rats, female and male rats were allowed to mate overnight until a vaginal plug was found, which was defined embryonic day 0.5 (E0.5). Sodium valproic acid (VPA; Sigma Aldrich, St Louis, MO, United States) was dissolved in 0.9% saline at a concentration of 250 mg ml^−1^, and pregnant rats received a single intraperitoneal (i.p.) injection of 600 mg kg^−1^ VPA or an equal volume of saline (VPA and SMPS groups treated with VPA and control groups treated with saline, respectively) on E12.5.

**Figure 1:**
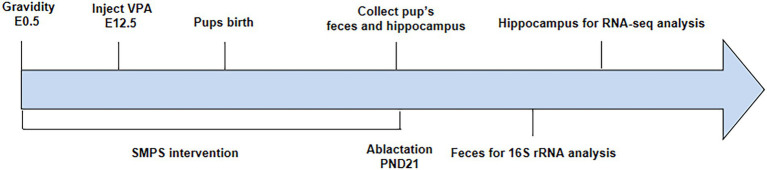
Schematic representation of the experimental procedure.

Pregnant rats injected with VPA were randomly divided into the VPA group and SMPS group (2 g/kg, 80% purity, Ruina, China), while pregnant rats injected with saline represented the control group (expressed as C). Each group included four pregnant rats, and each rat was kept in a single cage. The SMPS group received the SMPS intervention *via* drinking water on days E0.5-PND21, while the VPA and control groups received normal drinking water. Pregnant rats were individually housed and allowed to raise their own litters. The experimental procedure is shown in Figure 1.

### Collection of feces and hippocampus tissue

On PND 21, 8 pups from each group were individually placed into clean cages. Fecal pellets were quickly collected into sterile cryotubes after defecation, immediately transferred to liquid nitrogen, and subsequently stored at −80°C until 16S rRNA gene sequencing. Then, we sacrificed offspring from each group, rapidly placed the hippocampal tissue into sterile frozen storage tubes, transferred the samples to liquid nitrogen, and subsequently stored them at −80°C until RNA sequencing (RNA-seq).

### 16S rRNA gene sequencing and bioinformatics analysis

Total genomic DNA of each fecal sample (~100 mg) was extracted by an E.Z.N.A. Stool DNA Kit (OMEGA, United States). The DNA concentration and purity were monitored on 1% agarose gels. According to the concentration, DNA was diluted to 1 ng/μl with sterile water. 16S rRNA genes in distinct regions (16S V4) were amplified with specific primers (515F-806R) and barcodes. All PCR mixtures contained 15 μl of Phusion^®^ High-Fidelity PCR Master Mix (New England Biolabs), 0.2 μM of each primer and 10 ng target DNA, and the cycling conditions consisted of an initial denaturation step at 98°C for 1 min, followed by 30 cycles at 98°C (10 s), 50°C (30 s) and 72°C (30 s), and a final 5 min extension at 72°C.

The PCR products were mixed in equal proportions, and then the Qiagen Gel Extraction Kit (Qiagen, Germany) was used to purify the mixed PCR products. Sequencing libraries were generated with the NEBNext^®^ Ultra™ IIDNA Library Prep Kit (Cat No. E7645) following the manufacturer’s recommendations. The library quality was evaluated on a Qubit@ 2.0 Fluorometer (Thermo Scientific) and an Agilent Bioanalyzer 2100 system. Finally, the library was sequenced on an Illumina NovaSeq platform by Novogene Co., Ltd. (Tianjin, China).

Paired-end reads were merged using FLASH (Version 1.2.11). Quality filtering of the raw tags was performed using fastp (Version 0.20.0) software to obtain high-quality clean tags. The clean tags were compared with the reference database (Silva database https://www.arb-silva.de/for16S) using Vsearch (Version 2.15.0) to detect chimera sequences, which were then removed to obtain the effective tags. Alpha diversity indices, including Chao1, ACE, Shannon and Simpson indices, were calculated by Mothur. The results of a principal coordinates analysis (PCoA) based on the Bray–Curtis dissimilarity were analyzed by permutational multivariate analysis of variance (PERMANOVA) to determine the beta-diversity of different bacterial communities. Linear discriminant analysis effect size (LEfSe) was used to evaluate the influence of each taxon on differences between two groups.

### RNA-seq and bioinformatics analysis

#### RNA quantification, library preparation and sequencing

Four out of eight bioinformatics samples per group were used to carry out transcriptomic experiments. The RNA integrity of each group’s hippocampal tissue was assessed using the RNA Nano 6000 Assay Kit of the Bioanalyzer 2100 system (Agilent Technologies, CA, United States). The RNA integrity and DNA contamination of the samples were analyzed by agarose gelelectrophoresis using Agilent 2100 bioanalyzer (Agilent Technologies, Inc., United States). The concentration of RNA, the ratio of OD260/280 and OD260/230 was determined through Nano Drop spectrophotometer (Thermofsher Scientific Inc., MA, United States). The preparation of cDNA library and RNA sequencing was performed at Novogene Co., Ltd. (Tianjin, China).

#### Data preprocessing, mapping, and quantification

Raw data (raw reads) in fastq format were first processed through in-house Perl scripts. In this step, clean data (clean reads) were obtained by removing reads containing adapters and poly-N sequences and low-quality reads from the raw data. At the same time, the Q20, Q30, and GC contents of the clean data were calculated. All downstream analyses were based on clean high-quality data.

Reference genome and gene model annotation files were downloaded from the genome website directly. The index of the reference genome was built using HISAT2 v2.0.5, and paired-end clean reads were aligned to the reference genome using HISAT2 v2.0.5. We selected Hisat2 as the mapping tool because Hisat2 can generate a database of splice junctions based on the gene model annotation file and thus a better mapping result than other nonsplice mapping tools. The mapped reads of each sample were assembled By StringTie (v1.3.3b) in a reference-based approach. StringTie uses a novel network flow algorithm as well as an optional *de novo* assembly step to assemble and quantitate full-length transcripts representing multiple splice variants for each gene locus.

Feature Counts v1.5.0-p3 was used to count the read numbers mapped to each gene. Then, the FPKM of each gene was calculated based on the length of the gene and read count mapped to this gene. FPKM, the expected number of Fragments Per Kilobase of transcript sequence per Millions base pairs sequenced, considers the effect of sequencing depth and gene length for the read count at the same time and is currently the most commonly used method for estimating gene expression levels. All RNA-seq raw data have been submitted to Sequence Read Archive (accession number PRJNA870709; https://www.ncbi.nlm.nih.gov/bioproject/PRJNA870709).

#### Differential expression analysis

Prior to the differential gene expression analysis, for each sequenced library, the read counts were adjusted by the edge R program package through one scaling normalized factor. Differential expression analysis of the two conditions was performed using the edge R package (3.22.5). The *p*-values were adjusted using the Benjamini & Hochberg method. The *p*-value of 0.05 and an absolute fold change of 2 were set as the thresholds for significant differential expression.

#### Enrichment and pathways

Gene Ontology (GO) enrichment analysis of differentially expressed genes was implemented by the clusterProfiler R package, in which gene length bias was corrected. GO terms with *p*-values <0.05 were considered significantly enriched by differentially expressed genes. KEGG is a database resource for understanding the high-level functions and utilities of biological systems, such as cells, organisms and ecosystems, based on molecular-level information, especially large-scale molecular datasets generated by genome sequencing and other high-throughput experimental technologies.^1^ We used the clusterProfiler R package to test the statistical enrichment of differentially expressed genes in KEGG pathways.

### Statistical analysis

The data were analyzed using SPSS 18.0 (SPSS Inc., Chicago, IL, United States). All data are represented as the mean ± SD. Statistical analyses of multiple-group comparisons were performed by one-way ANOVA and PERMANOVA. Statistical significance was set at *p <* 0.05.

## Results

### Bacterial taxonomic profiles

**Figure 2:**
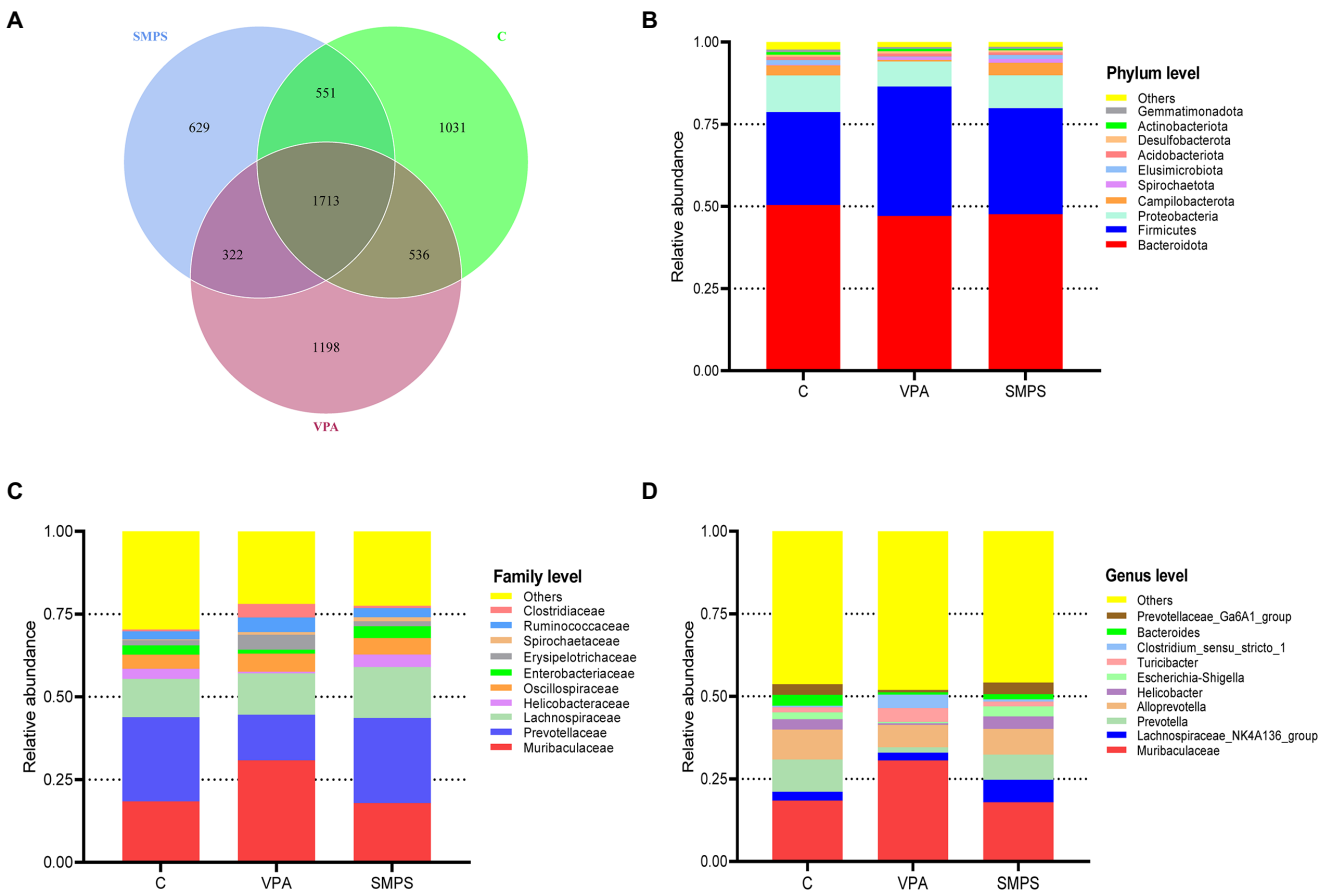
Characteristics of the gut microbiota among the SMPS, VPA and C groups (*n* = 8 per group). **(A)** Venn diagram for these groups. The number of mutual ASVs in the groups. Top 10 species at the **(B)** phylum level, **(C)** family level, and **(D)** genus level. (SMPS, SMPS intervention group; VPA, VPA induced autism model group; C, control group).

To detect the effect of SMPS on VPA-treated model rats, fecal samples were analyzed by 16S rRNA gene sequencing. A total of 2,561,178 raw PEs were obtained from the three groups (including 24 samples). ASV annotations were performed for all samples (41 at the phylum level, 104 at the class level, 245 at the order level, 391 at the family level, 650 at the genera level, 187 at the species level), and the number of intersecting ASVs differed among these groups (Figure 2A). Moreover, the relative abundances of the top 10 species at the phylum, family and genus levels are shown in Figures 2B–D. It showed that the SMPS could change the relative abundance of taxa composition at the family and genus levels, and the SMPS group was similar to the control group. Compared to VPA group, the relative abundance of *Muribaculaceae* was decreased in the SMPS group at the family level. In contrast, the relative abundance of *Prevotellaceae* was increased after SMPS intervention. In addition, SMPS group showed an increased relative abundance of *Lachnospiraceae_NK4A136_group* and *Helcobacter* compared with VPA group at genus level. However, the relative abundance of *Turicibacter* was decreased in the SMPS group, and similar to that of the control group. There was statistically significant differences in the ratio of *Bacteroidetes* to *Firmicutes* (B/F) among SMPS, VPA and C groups at the phylum level (*F*_(2,21)_ = 3.911, *p* = 0.036). The B/F ratio of VPA group was lower than that of C group (1.22 ± 0.40 & 1.83 ± 0.38, *p* = 0.011), whereas it was elevated after SMPS intervention (*p* > 0.05).

### Alpha and beta diversity analyses

**Figure 3:**
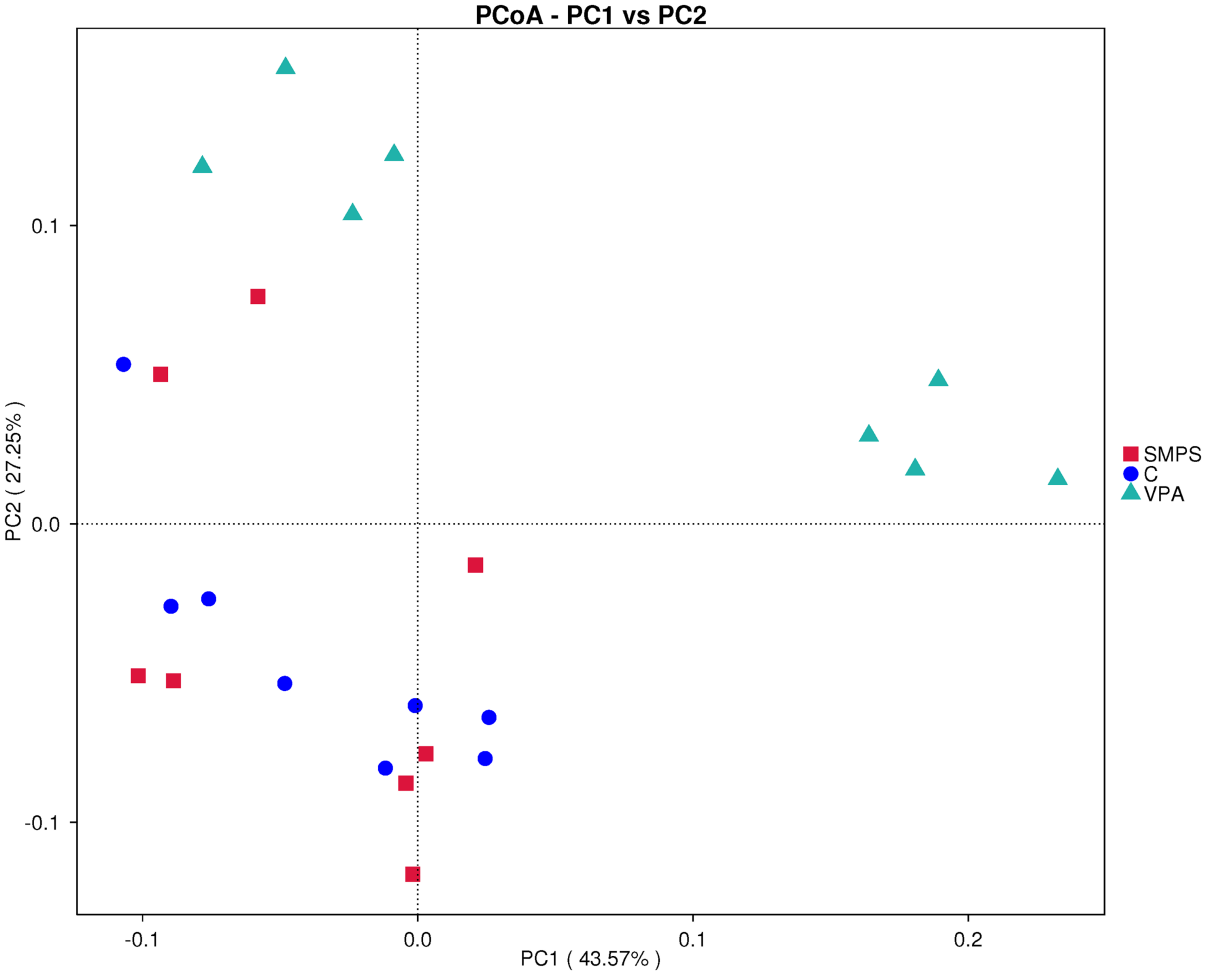
Beta diversity analysis for PCoA chart (*n* = 8, per group).

The α-diversity analysis showed significant differences in the Pielou (*F*_(2,21)_ = 13.158, *p* < 0.001) and Simpson indices (*F*_(2,21)_ = 7.602, *p* = 0.003) among these groups by one-way ANOVA. Then, through the LSD test, we found that the SMPS group was significantly different from the VPA group (*p*_(pielou)_ < 0.001, *p*_(simpson)_ = 0.001; Supplementary Figure S1). Beta diversity analyses provide a comparative analysis of the microbial community composition of different groups. PCoA (weighted UniFrac) was used after the ASV selection-based bacterial taxonomy analysis, which showed that the overall composition of GM was significantly different between the SMPS and VPA groups (*R* = 0.466, *p* = 0.004; Figure 3); however, the composition of GM in the SMPS group was not different from that of the controls (*R* = 0.051, *p* = 0.269). Based on above, it indicated that SMPS intervention had an effect on the composition of the gut microbiota in the VPA-induced model rats, and made the composition of gut microflora approached that of the control group.

### Linear discriminant analysis

**Figure 4:**
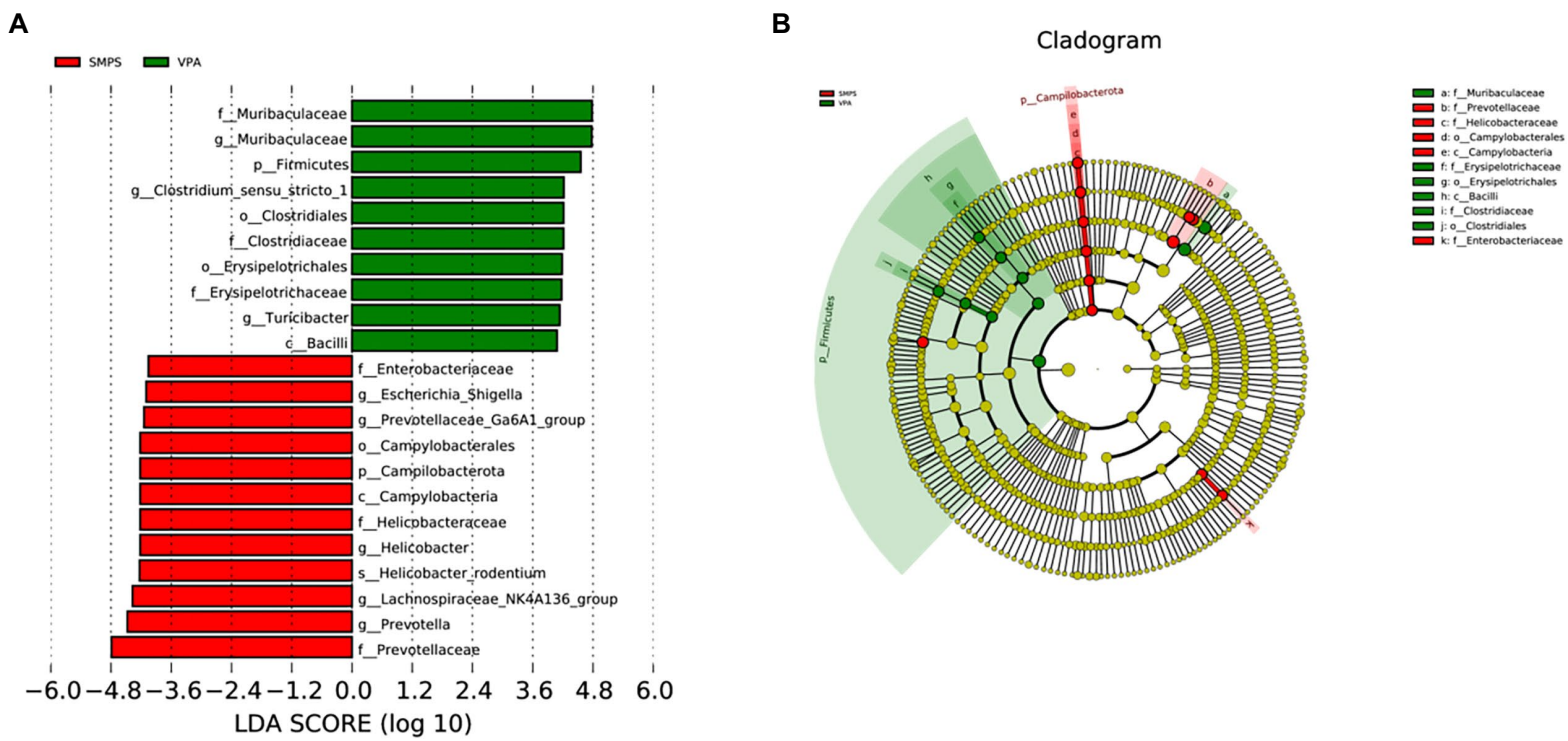
Significant bacterial taxa between the SMPS and VPA groups (*n* = 8 per group). **(A)** Cladogram based on the linear discriminant analysis effect size (LEfSE) method, the statistical significance cutoff: absolute linear discriminant analysis (LDA) score log10 ≥ 2.0; **(B)** Linear discriminant analysis (LDA) coupled with LEfSE between the SMPS and VPA groups.

The LDA effect size results revealed that *Enterobacteriaceae*, *Escherichia_Shigella*, *Prevbtellaceae_Ga6A1_group*, *Campylobacterales*, *Campilobacterota*, *Campylobacteria*, Helicbbacteraceae, *Helicobacter*, *Helicobacter_rodentium*, *Lachnospiraceae_NK4A136_group*, *Prevotella*, and Prevotellaceae were characteristic taxa in the SMPS group, while *Muribaculaceae, Firmicutes, Clostridium_sensu_stricto_1*, *Clostridiales, Clostridiaceae, Erysipelotrichales, Erysipelotrithaceae*, *Turicibacter* and *Bacilli* were enriched in the VPA group (Figure 4). The linear discriminant analysis showed a clear alteration of microbiota characterized by higher *Prevotellaceae* and *Lachnospiraceae_NK4A136_group* levels in the SMPS group (LDA score > 4). However, *Muribaculaceae* and *Firmicutes* levels were significantly reduced after SMPS intervention. At the same time, we found that *Lachnospiraceae_NK4A136_group* was the characteristic taxa in the SMPS group compared with C group, and *Bacteroides_sartorii*, *Bacteroides and Bacteroidanceae* were enriched in the C group (LDA score > 3.5).

### RNA-seq for differential expression analysis

**Figure 5:**
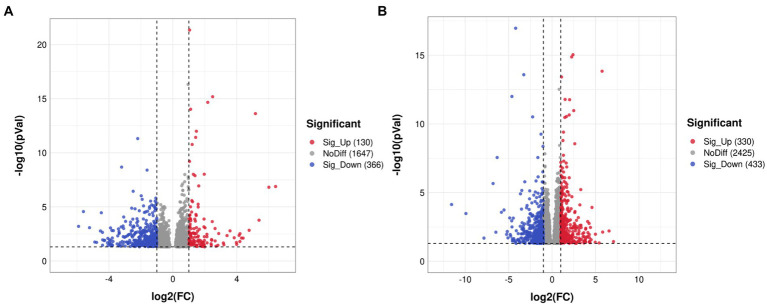
Volcano plots showing distribution trends for differentially expressed genes (*n* = 4 per group). **(A)** SMPS and VPA groups; **(B)** SMPS and C groups. Red and blue dots indicate up- and downregulated DEGs, respectively; gray dots indicate that the genes were not differentially expressed.

**Figure 6:**
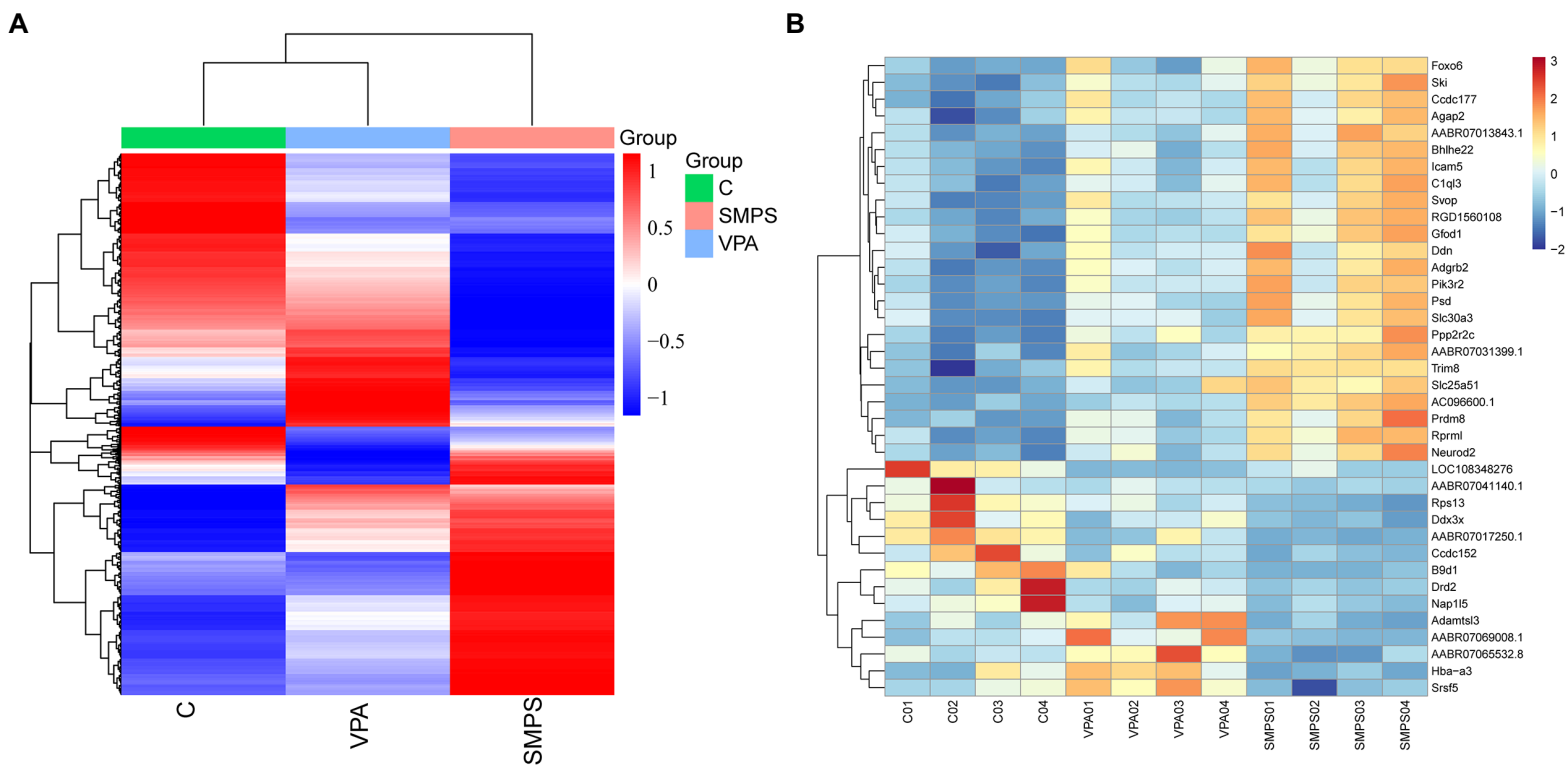
Cluster heatmaps among the SMPS, VPA and C groups (*n* = 4 per group). **(A)** Hierarchical clustering heatmap of DEGs among the three groups; **(B)** Clustering heatmap showed 38 common DEGs among the three groups.

In total, 187,012,228, 188,078,284 and 186,294,964 raw reads were produced from the SMPS, VPA and control groups, respectively. The Q20% was above 97% among these groups, and the GC percentages of the clean reads were 48.85%, 50.16%, and 49.16% in the SMPS, VPA and control groups, respectively. In addition, 158,025,892, 156,405,535, and 157,347,614 reads were mapped to the rat genome in the groups. We finally obtained 14,094, 14,397, and 14,230 genes in the SMPS, VPA and control groups, respectively. A total of 496 genes were identified as DEGs from the SMPS and VPA groups, of which 130 were up-regulated and 366 were down-regulated (Figure 5A). The top five up-regulated genes were *Ebna1bp2* (log_2_FC = 6.43, padj = 8.73E-05), *Nudt16l1* (log_2_FC = 1.97, padj = 1.02E-05), *Cebpb* (log_2_FC = 1.62, padj = 1.15E-07), *Pbld1* (log_2_FC = 1.59, padj = 2.74E-03) and *Moap1* (log_2_FC = 1.58, padj = 0.02), whereas the top five down-regulated genes were *Timm17b* (log_2_FC = −5.60, padj = 4.99E-03), *Nxnl2* (log_2_FC = −3.12, padj = 0.04), *Cnksr1* (log_2_FC = −3.08, padj = 0.01), *Bhmt* (log_2_FC = −2.65, padj = 0.04) and *Bnc2* (log_2_FC = −2.51, padj = 0.02). Compared with the C group, 763 DEGs were identified in the SMPS intervention group, of which 330 DEGs were up-regulated and 433 DEGs were down-regulated (Figure 5B). The top five up-regulated genes were *Esm1* (log_2_FC = 4.59, padj = 7.97E-03), *Tfap2c* (log_2_FC = 3.20, padj = 6.06E-03), *Gdf15* (log_2_FC = 3.02, padj = 0.02), *Pigg* (log_2_FC = 2.64, padj = 0.01) and *Cd3e* (log_2_FC = 2.61, padj = 0.01), whereas the top five down-regulated genes were *Nupr1* (log_2_FC = −5.83, padj = 0.01), *Chat* (log_2_FC = −4.65, padj = 0.03), *B9d1* (log_2_FC = −4.62, padj = 2.06E-09), *Haus1* (log_2_FC = −3.52, padj = 9.71E-03) and *Ngfr* (log_2_FC = −3.44, padj = 0.04). The expression of DEGs among the three groups is shown in Figure 6A. The total of 38 DEGs co-expressed in the SMPS, VPA and control groups are displayed in Figure 6B.

### Go pathway analyses

**Figure 7:**
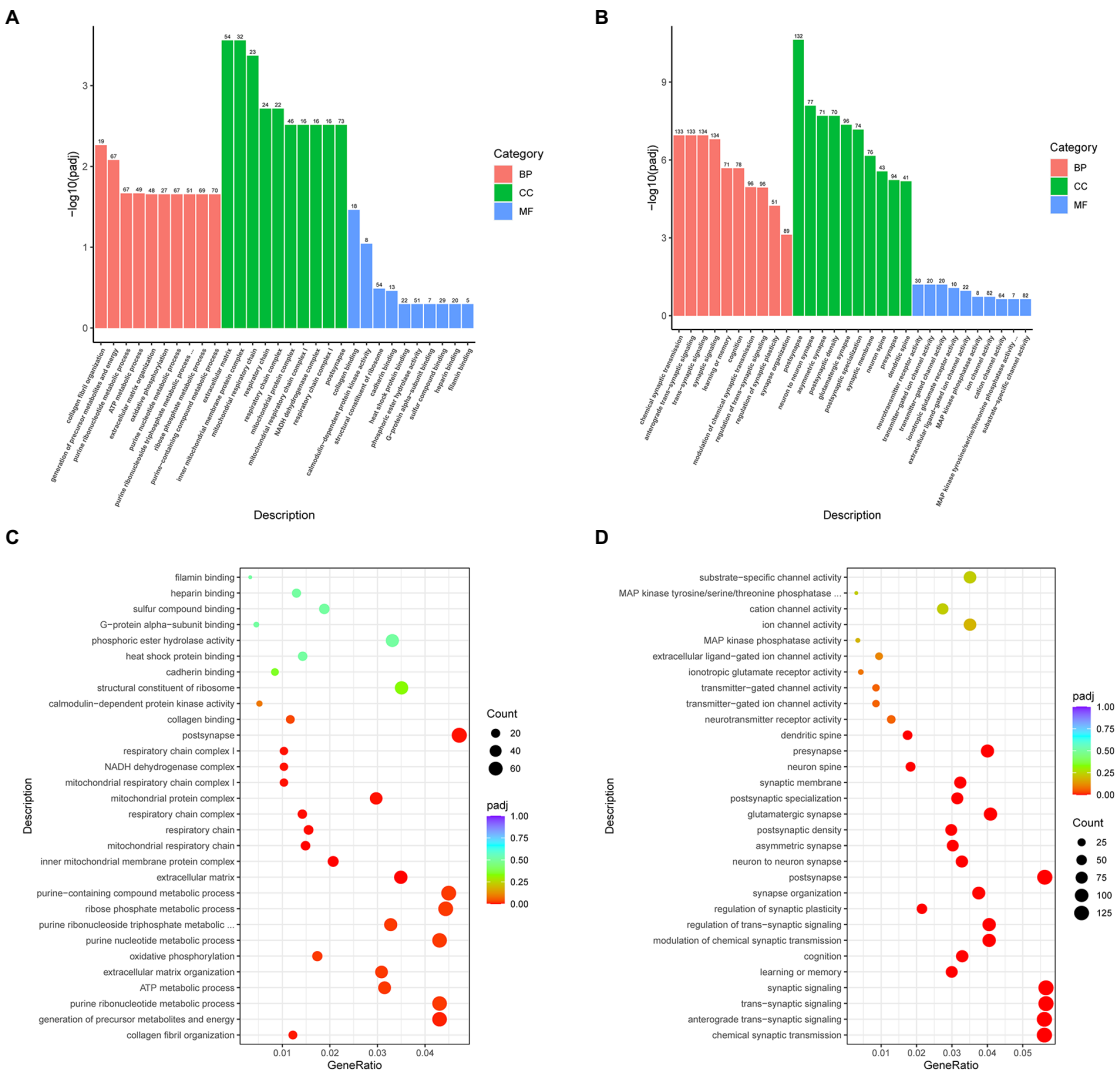
Bar charts and dot plots of the main GO term enrichment analysis (*n* = 4 per group). Bar chart of the comparison showing the 30 most significant terms between the **(A)** SMPS and VPA groups and **(B)** SMPS and C groups (BP, biological process; CC, cell component; and MF, molecular function); and dot plot of the comparison between the **(C)** SMPS and VPA groups and **(D)** SMPS and C groups, with every dot representing one pathway. The size and color of each circle represented the gene number in this pathway and *p*-value, respectively.

The functions of the DEGs were analyzed based on the GO database, and we obtained 653 significantly different GO terms between the SMPS and VPA groups, including biological processes (548 subclasses), cellular components (65 subclasses), and molecular functions (40 subclasses). The top five biological process terms were collagen fibril organization, precursor metabolite and energy generation, purine ribonucleotide metabolism, ATP metabolic process and extracellular matrix organization. In addition, the extracellular matrix, inner mitochondrial membrane protein complex, mitochondrial respiratory chain, respiratory chain and respiratory chain complex were dominant among the cellular components. The top five molecular function terms for GO analysis were collagen binding, calmodulin-dependent protein kinase activity, structural constituent of ribosome, cadherin binding and heat shock protein binding, which are shown in Figures 7A,C. Meanwhile, we got 817 significantly different GO terms between the SMPS and C groups, including biological processes (639 subclasses), cellular components (99 subclasses), and molecular functions (79 subclasses). The results of GO BP were significantly enriched in chemical synaptic transmission, anterograde trans-synaptic signaling, trans-synaptic signaling, synaptic signaling and learning or memory. The postsynapse, neuron to neuron synapse, asymmetric synapse, postsynaptic density and glutamatergic synapse were the most strongly represented cellular components. The top five molecular function terms for GO analysis were neurotransmitter receptor activity, transmitter-gated ion channel activity, transmitter-gated channel activity, ionotropic glutamate receptor activity and extracellular ligand-gated ion channel activity (Figures 7B,D).

### Integrated enrichment analysis of 16S rRNA and transcriptomics profiles

**Figure 8:**
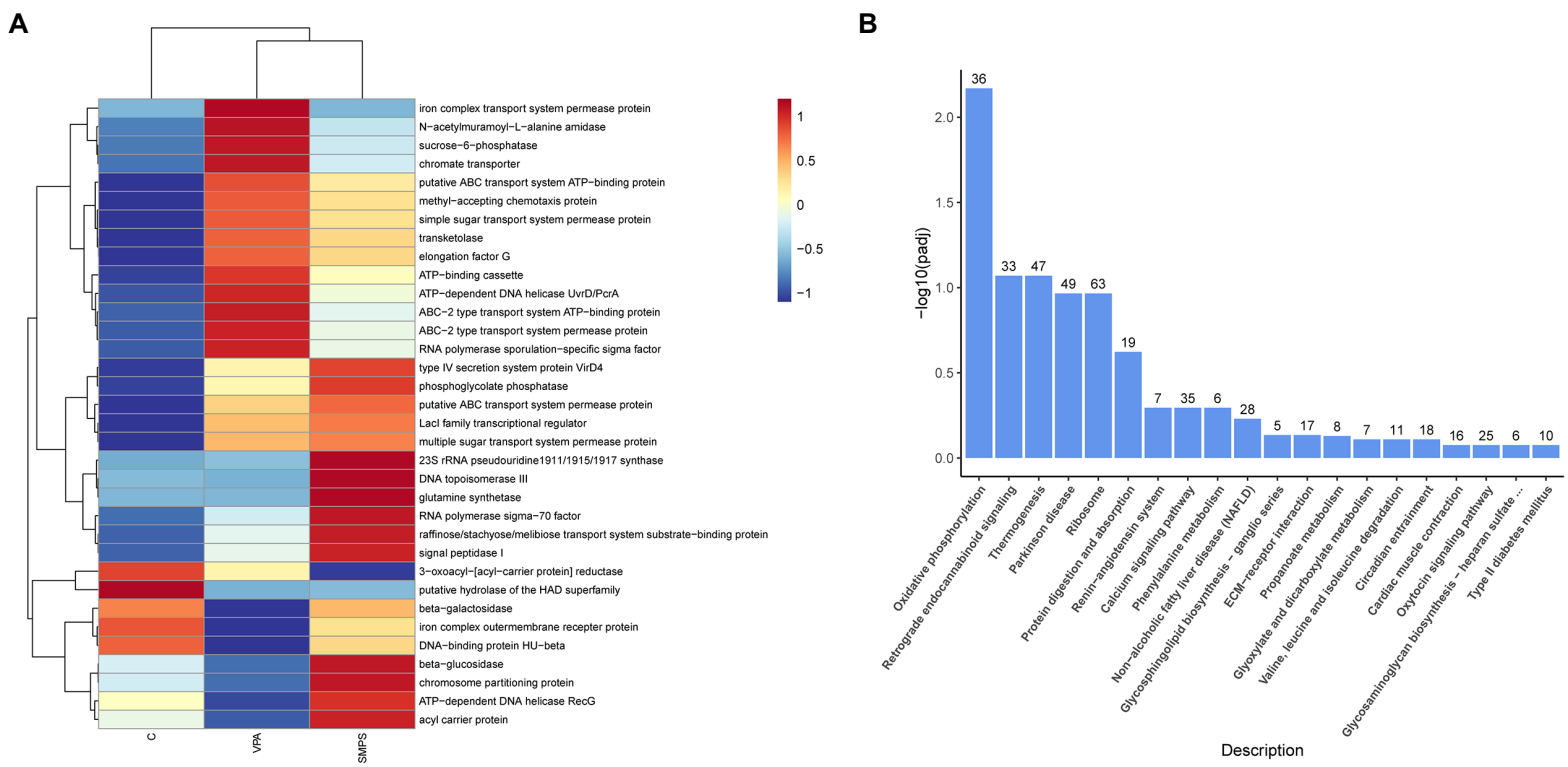
The KEGG analysis for transcript and 16S rRNA profiles. **(A)** Heatmap of functional annotation and abundance information for 16S rRNA profile among SMPS, VPA and C groups; **(B)** Bar chart of the enrichment pathways between SMPS and VPA groups.

In the 16S rRNA profile, based on the functional annotation and abundance information of the samples in the database, the 34 abundance functions of GM in each group were selected to heatmap and clustered from different functional levels (Figure 8A). Subsequently, by KEGG pathway analysis, we found that abundance functions were enriched in metabolic pathways, biosynthesis of secondary metabolites, starch and sucrose metabolism, biosynthesis of amino acids, microbial metabolism in diverse environments, galactose metabolism, other glycan degradation, GABAergic synapse, etc.

For the KEGG pathway analysis of transcriptomics profile, DEGs associated with SMPS relative to the VPA group were mostly enriched in oxidative phosphorylation, retrograde endocannabinoid signaling, thermogenesis, Parkinson disease, ribosome, protein digestion and absorption, renin-angiotensin system, calcium signaling pathway, phenylalanine metabolism, glycosphingolipid biosynthesis-ganglio series, and propanoate metabolism, etc. (Figure 8B; Supplementary Table S1). In addition, after the SMPS intervention, differentially expressed genes were enriched in the oxidative phosphate signaling pathway, including 22 upregulated genes (Uqcrb, Ndufa6, Ndufa3, etc.) and 14 downregulated genes (Mt-nd3, Cox7a2l2, Mt-cyb, etc.). We also found that both upregulated and downregulated genes were mostly enriched in the ribosome pathway. Furthermore, compared with the controls, DEGs induced by SMPS were enriched in the calcium signaling pathway, dilated cardiomyopathy, adrenergic signaling in cardiomyocytes, vascular smooth muscle contraction, dopaminergic synapse, endocytosis, and aldosterone synthesis and secretion, etc. (Supplementary Table S2).

Based on the above findings, it was possible that SMPS intervention affected biological metabolism (including starch and sucrose metabolism, biosynthesis of amino acids, galactose metabolism and other glycan degradation pathways) through regulating intestinal flora. For this, it might play a role in signaling pathways related to neural function in the VPA induced model rats, such as oxidative phosphorylation, retrograde endocannabinoid signaling, ribosome, renin-angiotensin system, calcium signaling pathway, glycosphingolipid biosynthesis-ganglio series, and propanoate metabolism, etc.

## Discussion

In this study, after the SMPS intervention (from pregnancy to lactation) of VPA-injected pregnant rats, the diversity and structure of the GM in their pups were changed, especially the relative abundance of *Prevotellaceae* and *Lachnospiraceae_NK4A136_group*. The finding confirmed the supporting effect of plant polysaccharides on the intestinal microbiota. To authenticate whether it has initiated the gut-brain axis, we performed a transcriptomic analysis of the offspring’s hippocampal tissue. Interestingly, the overall findings showed that 496 genes were differentially expressed after induction by SMPS compared with the VPA group. Moreover, the GO analysis indicated that these DEGs were mainly involved in collagen fibril organization, precursor metabolite and energy generation, and purine ribonucleotide metabolic processes. The KEGG analysis showed that both upregulated and downregulated genes were mostly enriched in the ribosome pathway. The above findings provide scientific evidence to elucidate the relationship between ASD and the microbiome-gut-brain axis.

Numerous studies have shown that plant polysaccharides can improve the intestinal flora and regulate the body’s immune system (Gudi et al., 2019; Youn et al., 2020; Guo et al., 2021). It is worth noting that a large number of studies have shown a strong association between the etiology of ASD and its disturbance of immune function (Meltzer and Van de Water, 2017; Garcia-Gutierrez et al., 2020). Furthermore, the gut microbiome is an integral part of the gastrointestinal tract and involved in the absorption and digestion of polysaccharides in food, and it simultaneously influences human health and the occurrence of disease (Cockburn and Koropatkin, 2016). The GM has a strong connection with the central nervous system (Suganya and Koo, 2020). ASD, as a heterogeneous, behaviorally defined disorder, is a neurodevelopmental disease, and the observed behavioral and cognitive features are associated with pervasive atypicalities in the central nervous system (Shuid and Jayusman, 2020). Early life is a critical period for the formation and development of the nervous system, especially from maternal intervention (Foong and Hung, 2020). The nutrients and risk factors in early life are all associated with the mother, and they play an important role in the GM and neural development of the offspring (Buffington et al., 2016; Wang et al., 2017).

To date, many studies have reported alterations in the GM of ASD children and autistic animal models (Schneider and Przewłocki, 2005; Ma et al., 2019; Wong et al., 2021). However, due to the influence of the dietary structure and habits of ASD children, considerable heterogeneity is observed in the changes in the intestinal flora (Liu et al., 2019; Iglesias-Vázquez et al., 2020; Zou et al., 2020). Exposure to valproic acid (VPA) during pregnancy has been demonstrated to increase the risk of autism in children (Nicolini and Fahnestock, 2018). Moreover, rodents prenatally exposed to VPA display behavioral phenotypes characteristic of the human condition. At the same time, VPA-induced autism model rats are highly similar with ASD patients in terms of genetics, physiology and metabolism and have been widely used as an ideal model for studying the etiology of autism (Tartaglione et al., 2019; Mehra et al., 2022). In this study, the SMPS intervention was continuously administered to pregnant rats from pregnancy to lactation, and pup feces were collected at the end of lactation (PND21). In this way, the effect of SMPS on the VPA-induced intestinal flora of model rats can be more accurately explored. Through 16S rRNA gene sequencing analysis, we found that the richness and diversity of the GM showed significant differences among the SMPS, VPA and control rats, which is consistent with the findings of a previous study (Gu et al., 2021). A recent report found that the richness and diversity of gut microbiota did not differ between VPA and control groups, while the overall composition of gut microbiota was significantly different (Gu et al., 2022). In addition, the PCoA results revealed that the overall composition of the GM was different between the SMPS and VPA groups. At the phylum level, the taxa composition of the three groups mainly contained *Bacteroidota, Firmicutes* and *Proteobacteria*; however, the ratio of each group was different. The relative abundance of *Firmicutes* was decreased after SMPS intervention. A previous study based on gut microbiota of the ASD population found that the relative abundance of *Firmicutes* in the ASD group was increased, which is consistent with the findings for the VPA-induced model rats in the present study (Strati et al., 2017). Moreover, the ratio of *Bacteroidetes* to *Firmicutes* in the VPA group was lower than that of C group, which is agreement with the provious studies of ASD children (Tomova et al., 2015; Strati et al., 2017). This suggests that the gut microbiota characteristics of the VPA-induced model rats are similar to those of ASD children. It was worth noted that the B/F ratio was elevated after SMPS intervention compared to the VPA group. For this, it revealed that SMPS treatment ameliorated the intestinal microbiota of VPA induced rats.

To find key biomarkers in the gut microbiota of the SMPS and VPA rats, we conducted linear discriminant analysis, which is an analytical tool for discovering and interpreting high-dimensional biological identifiers (Segata et al., 2011). In the current study, the difference of taxa was significant between SMPS and VPA groups, especially in *Prevotellaceae* and *Lachnospiraceae_NK4A136_group*. One study has reported that *Prevotellaceae* was the specific causal microbe taxa for autism spectrum disorder (Ni et al., 2021). In comparative studies between ASD and normal children, the relative abundance of *Prevotellaceae* in the ASD children was significantly lower than that of control children, whereas our study indicated that SMPS intervention was able to increase the abundance of *Prevotellaceae* (Pulikkan et al., 2018; Qiao et al., 2018; Sun et al., 2019). Moreover, the change of *Lachnospiraceae_NK4A136_group* is significantly correlated with enhanced gut barrier function (Ma et al., 2020). Thus, SMPS may have an important role for regulating the dysbiosis of intestinal flora in the ASD. These findings were consistent with the effects of various plant polysaccharides on human and animal intestinal microbiota (Wang et al., 2020; Zhu et al., 2020; Cui et al., 2021; Sang et al., 2021). In addition, the functional enrichment analysis of 16S rRNA profile suggested that SMPS intervention affected biological metabolism (including starch and sucrose metabolism, biosynthesis of amino acids, galactose metabolism and other glycan degradation pathways) through regulating intestinal flora. Interestingly, the recent literature indicated that the gut microbiota had effect on brain functions through endocrine and metabolic pathways and the enteric network, especially in the onset and maintenance of neurodevelopment and neurodegenerative disorders (Marazziti et al., 2021). ASD is a neurodevelopmental disease. To further explore whether SMPS intervention regulates the gut-brain axis through the gut microbiota, we performed transcriptome sequencing of the hippocampal tissue.

The early stage of life is a critical period for the formation and development of the nervous system (Cisneros-Franco et al., 2020). In this study, SMPS intervention began in pregnancy and continued to the juvenile weaning period, which is the key period of brain and nervous system development in the young rats, and the nutritional source was mainly derived from the mothers. Through the transcriptomic sequencing results, we found 496 DEGs between the SMPS and VPA groups. Subsequently, GO analysis showed that these DEGs were mainly involved in collagen fibril organization, the precursor metabolite and energy generation, and purine ribonucleotide metabolic processes. Meanwhile, the KEGG analysis showed that these DEGs were enriched in oxidative phosphorylation, retrograde endocannabinoid signaling, thermogenesis, Parkinson disease, ribosome, protein digestion and absorption, renin-angiotensin system, calcium signaling pathway, phenylalanine metabolism, glycosphingolipid biosynthesis-ganglio series, and propanoate metabolism. Based on the enrichment analysis, we focused more on oxidative phosphorylation and ribosomes, which were both enriched in the GO and KEGG analyses. Some studies have reported that ASD patients possess abnormal oxidative phosphorylation, which is associated with autistic behaviors (including social interaction, abnormal behavior, and verbal communication; Legido et al., 2013; Napoli et al., 2014; Singh et al., 2014). Many ASD patients also exhibit mitochondrial disease, and the oxidative phosphorylation process occurs precisely in the mitochondria; therefore, abnormal oxidative phosphorylation may induce impaired mitochondrial function, followed by oxidative stress, resulting in excess reactive oxygen species with certain neurotoxicity, which may be a possible cause of the occurrence of ASD (Martinez-Finley et al., 2013; Rose et al., 2018; Bjørklund et al., 2020; Manivasagam et al., 2020). After the SMPS intervention, differentially expressed genes were enriched in the oxidative phosphate signaling pathway, including 22 upregulated genes (Uqcrb, Ndufa6, Ndufa3, etc.) and 14 downregulated genes (Mt-nd3, Cox7a2l2, Mt-cyb, etc.). Development of the nervous system is carried out by complex gene expression programs that are regulated at both the transcriptional and translational levels. The production of ribosomes is essential for protein synthesis. Mutations in several ribosomal components and trans-acting ribosomal biogenesis factors result in neurodevelopmental syndromes that present with autism, intellectual deficits and/or progressive neurodegeneration (Hetman and Slomnicki, 2019). One study of autistic etiology found that mice lacking the Eif4g1 microexon, which functions as a translational brake by causing ribosome stalling, displayed deficits in social behavior, learning, and memory as well as altered hippocampal synaptic plasticity (Gonatopoulos-Pournatzis et al., 2020). Additionally, a previous report indicated that the individual copy number of ribosomal genes is a factor associated autism risk and severity (Porokhovnik, 2019). In this study, the differentially expressed genes enriched in the ribosomal pathway included 43 upregulated genes (Rps4x, Rpl21, Mrps10, etc.) and 20 downregulated genes (Rpl39, Rps4y2, Rps13, etc.). In the KEGG enrichment analysis results, propanoate metabolism pathway also attracted our attention, which included 8 DEGs. The animal research revealed that propionic acid-treated rats display ASD-like repetitive, perseverative, and antisocial behaviors and seizure. Moreover, the neurochemical changes were consistent with findings in ASD patients, including mitochondrial dysfunction (Witters et al., 2016). Propanoate metabolism pathway has bioactive effects on neurotransmitter systems, calcium release, fatty acid metabolism (Macfabe, 2013). It is similarity to the GO and KEGG enrichment results of this study, which provided a scientific basis for the association of propionate metabolic pathway and ASD.

## Conclusion

The above evidence strongly suggests that continuous SMPS intervention in the early life of autism model rats could change the diversity and composition of their GM and simultaneously affect the expression of a large number of genes. To date, this is the first study on the effects of plant polysaccharide intervention on the gut microflora and transcriptome of autism, which not only provides an important scientific basis for the role of the microbe-gut-brain axis in ASD research but also provides a new direction for the treatment of ASD.

## Data availability statement

The original contributions presented in the study are publicly available. This data can be found at: NCBI, PRJNA870709.

## Ethics statement

The animal study was reviewed and approved by Ethics Committee of Qiqihar Medical University (QMU-AECC-2021-62).

## Author contributions

XY: conceptualization, project administration, and writing original manuscript. JiyL: methodology and statistical analysis. YZ: methodology. NZ: methodology and supervision. JicL: writing–review and editing. All authors contributed to the article and approved the submitted version.

## Funding

This work was supported by the Natural Science Foundation of Heilongjiang Province (no. LH2020H131) and the National Natural Science Foundation of China (no. 82103869).

## Conflict of interest

The authors declare that the research was conducted in the absence of any commercial or financial relationships that could be construed as a potential conflict of interest.

## Publisher’s note

All claims expressed in this article are solely those of the authors and do not necessarily represent those of their affiliated organizations, or those of the publisher, the editors and the reviewers. Any product that may be evaluated in this article, or claim that may be made by its manufacturer, is not guaranteed or endorsed by the publisher.

## Footnotes

^1^ http://www.genome.jp/kegg/

## Supplementary material

SUPPLEMENTARY FIGURE S1 Alpha diversity analysis (n=8 per group). **(A)** Pielou index and **(B)** Simpson index.
