# Supplementary figures and images for: Effect of stigma maydis polysaccharide on the gut microbiota and transcriptome of VPA induced autism model rats
Source: Front Microbiol. 2022 Nov 4;13:1009502. doi: 10.3389/fmicb.2022.1009502 (PMC9672813; doi:10.3389/fmicb.2022.1009502)

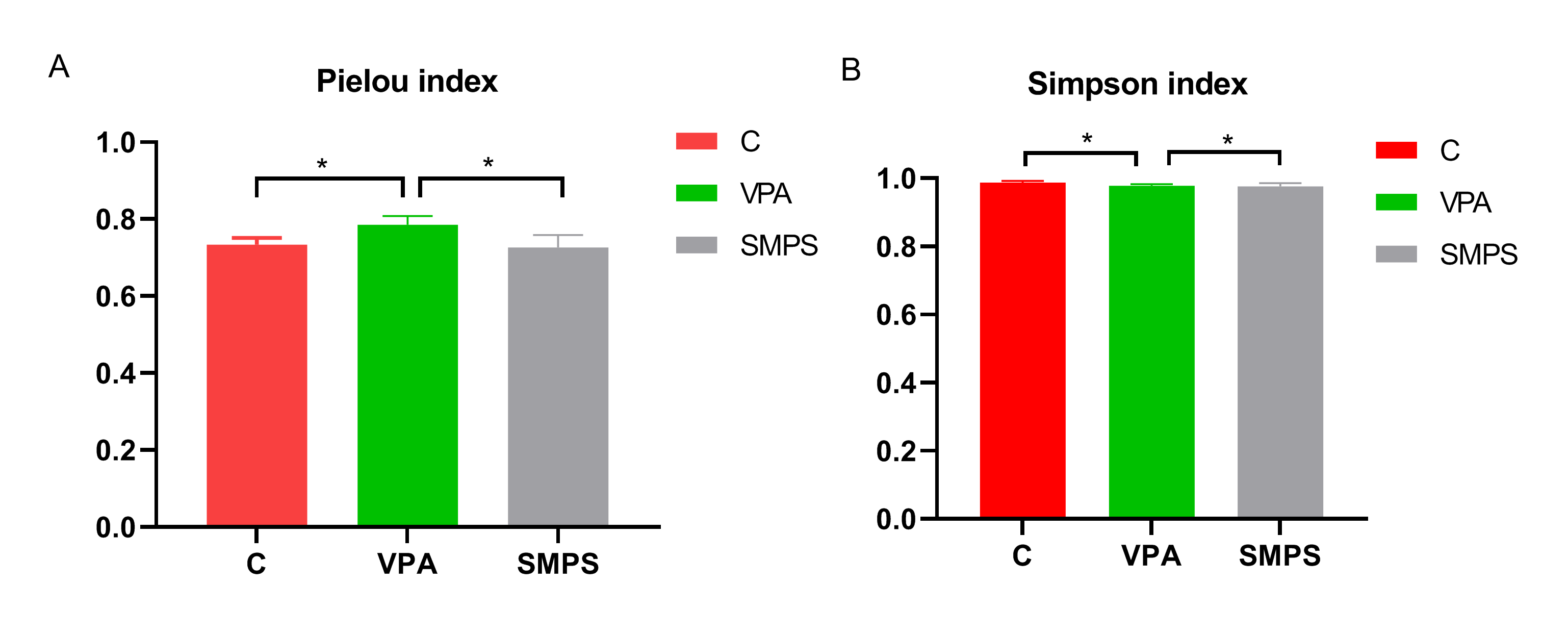

Supplement: SUPPLEMENTARY FIGURE S1 — Alpha diversity analysis (n=8 per group). (A) Pielou index and (B) Simpson index. [file Image_1.TIF]
