# Supplementary material for: Effect of stigma maydis polysaccharide on the gut microbiota and transcriptome of VPA induced autism model rats
Source: Front Microbiol. 2022 Nov 4;13:1009502. doi: 10.3389/fmicb.2022.1009502 (PMC9672813; doi:10.3389/fmicb.2022.1009502)
Supplement: Supplementary file 2 [file Data_Sheet_1.docx]

Table S1. The top ten enriched functional clusters of KEGG pathway between SMPS and VPA groups(n=4).

| **KEGG ID** | **Pathway description** | **Gene count** | ***P* value** | **Genes** |
| --- | --- | --- | --- | --- |
| rno00190 | Oxidative phosphorylation | 36 | 2.20E-05 | Uqcrb/AABR07015346.1/Ndufa6/Mt-nd3/Ndufa3/LOC100911417/Lhpp/LOC100363268/Ndufa1/Cox5b/Uqcrb/Ndufa7/Atp5f1e/LOC679739/Ndufb6/Cox5a/Atp5mc3/Ndufs8/Mt-nd4/AABR07011697.1/Ndufa11/Ndufs5/Cox7a2l2/Ndufb2/Mt-nd1/Mt-cyb/Atp5pd/AABR07046778.1/Atp5f1d/LOC684509/Cox4i1/Mt-atp6/Mt-nd2/Cox8a/Ndufs7/Atp6v1c2 |
| rno04723 | Retrograde endocannabinoid signaling | 33 | 0.00079668 | Ndufa6/Gng5/Mt-nd3/Gng4/Ndufa3/-/Adcy1/LOC100363268/Ndufa1/Kcnj6/Gabra3/Ndufa7/Adcy9/LOC679739/Gng10/Ptgs2/Ndufb6/Ndufs8/Mt-nd4/Ndufa11/Ndufs5/Adcy8/Ndufb2/Mapk14/Mt-nd1/Mapk8/AABR07046778.1/Cacna1c/Dagla/LOC684509/Mt-nd2/Gabra1/Ndufs7 |
| rno04714 | Thermogenesis | 47 | 0.000828851 | Uqcrb/AABR07015346.1/Ndufa6/Mt-nd3/Ndufa3/LOC100911417/Adcy1/Prkab1/LOC100363268/Ndufa1/Rps6ka3/Cox5b/Uqcrb/Ndufa7/Adcy9/Cox19/Atp5f1e/LOC679739/Slc25a29/Ndufb6/Cox5a/Atp5mc3/Ndufs8/Mt-nd4/Ndufa11/Ndufs5/Cox7a2l2/Adcy8/Ndufb2/Cox20/Mapk14/Mt-nd1/Pnpla2/Mt-cyb/Atp5pd/AABR07046778.1/Atp5f1d/Prkg2/LOC684509/Cox4i1/Mt-atp6/Mt-nd2/Creb1/Cox8a/Ndufs7/Dpf1/Smarcd1 |
| rno05012 | Parkinson disease | 49 | 0.00165836 | Uqcrb/AABR07015346.1/Ndufa6/Mt-nd3/Ndufa3/Slc18a1/LOC100911417/Ube2g2/Camk2b/LOC100363268/Ndufa1/Xbp1/Cox5b/Camk2d/Uqcrb/Ndufa7/Atp5f1e/LOC679739/Drd2/Camk2a/Drd1/Ndufb6/Tuba8/Cox5a/Atp5mc3/Ndufs8/Hspa5/Mt-nd4/Ndufa11/Ndufs5/Cox7a2l2/Psmc3/Ndufb2/Psmb5/Mt-nd1/Mt-cyb/Mapk8/Psmb2/Atp5pd/AABR07046778.1/Park7/Atp5f1d/LOC684509/Cox4i1/Mt-atp6/Tuba4a/Mt-nd2/Cox8a/Ndufs7 |
| rno03010 | Ribosome | 63 | 0.001755005 | Rps4x/-/AABR07073039.1/LOC100365810/AABR07049516.1/-/LOC100364457/LOC108350501/Rps28/Rpl37/LOC100360117/AABR07067829.1/Rpl39/RGD1562402/AABR07039448.1/LOC100910528/Rpl21/AABR07048031.1/LOC103693375/LOC498555/AABR07027322.1/AABR07031089.1/Rps3/Rps4y2/LOC100361933/Rps13/AABR07015294.1/Rpl10l/AABR07071891.1/AABR07034639.1/AABR07064000.1/LOC100360573/AABR07060610.1/AABR07017250.1/LOC690468/AABR07044509.1/AABR07050545.1/Mrps10/Rpl36/Rpl18a/Rpl36a/Rps21/LOC108349682/AABR07026317.1/AC128960.1/AABR07064702.1/AABR07072207.1/LOC108352650/Mrps12/Rpl37a/Mrpl27/Rpl13a/AC128859.4/Rpl10/Rpl30/LOC100362027/Rpl13/AABR07049821.1/Mrpl17/Rps25/AABR07064810.1/Rpl14/AABR07052430.1 |
| rno04974 | Protein digestion and absorption | 19 | 0.004641214 | Slc7a8/Dpp4/Col9a1/Eln/Col6a2/Col5a1/Slc9a3/Col14a1/Col27a1/Col6a1/AABR07068316.1/Ace2/Col1a1/Prcp/Slc8a3/Col12a1/Col4a5/Col4a1/Slc3a2 |
| rno04614 | Renin-angiotensin system | 7 | 0.013720328 | Thop1/Enpep/Mas1/Ace2/Lnpep/Prcp/Anpep |
| rno04020 | Calcium signaling pathway | 35 | 0.013890187 | Phkg1/Gna11/Adcy1/F2r/Adra1d/Camk2b/Cckbr/Itpka/P2rx4/Orai2/Camk2d/Adcy9/Camk2a/Drd1/Atp2b4/Grin2a/Adra1a/Htr2a/P2rx7/Ntsr1/Adcy8/Htr2c/Cacna1c/Phka1/Ppp3ca/Slc8a3/Sphk2/Ednra/Adra1b/Ptk2b/Asph/Phkb/Ryr2/Pde1c/Camk1g |
| rno00360 | Phenylalanine metabolism | 6 | 0.014815434 | Mif/Aldh3b1/Aldh3a1/Pah/LOC688778/Hpd |
| rno04932 | Non-alcoholic fatty liver disease (NAFLD) | 28 | 0.019133489 | Uqcrb/AABR07015346.1/Ndufa6/Ndufa3/Prkab1/LOC100363268/Ndufa1/Xbp1/Cox5b/Uqcrb/Ndufa7/LOC679739/Cebpa/Ndufb6/Cox5a/Ndufs8/Ndufa11/Ndufs5/Cox7a2l2/Ndufb2/Mt-cyb/Mapk8/LOC684509/Cox4i1/Cox8a/Ndufs7/Pik3r2/Adipor2 |

Table S2. The top ten enriched functional clusters of KEGG pathway between SMPS and control groups(n=4).

| **KEGG ID** | **Pathway description** | **Gene count** | ***P* value** | **Genes** |
| --- | --- | --- | --- | --- |
| rno04020 | Calcium signaling pathway | 58 | 7.22E-05 | Gna11/Adcy1/Camk2d/Camk2b/Drd1/P2rx4/P2rx7/Phkg1/Slc8a3/Calml4/Slc8a2/Cd38/Orai2/Cacna1h/Htr2a/Ntsr1/Itpka/Ptk2b/Camk2a/Plcd3/Mylk/LOC103694905/Chrm2/Plcb4/Ednra/Pln/Sphk2/Cacna1f/Gnas/Erbb4/Casq2/Adora2a/Adcy8/Asph/Cxcr4/Adra1a/Cacna1c/Cacna1i/-/Ednrb/Atp2b3/Gnal/Trdn/Calm2/Agtr1a/Htr2c/Slc25a4/Cacna1a/Orai1/Adra1d/Grin1/Pde1c/Stim2/Adra1b/Adcy4/Chrm1/Prkacb/Cacna1d |
| rno05414 | Dilated cardiomyopathy (DCM) | 31 | 0.000121544 | Cacng8/Adcy1/Slc8a3/Slc8a2/Cacnb4/Cacnb3/Cacnb1/Itga8/Itgb1/AABR07052585.2/Pln/Itgb4/Cacna1f/Gnas/Adcy8/Cacna2d2/-/Cacna1c/Cacng5/Itga6/Itga1/Mybpc3/Tgfb3/Itga11/Adcy4/Itga5/Itgb6/Prkacb/Cacna1d/Des/Sgcb |
| rno04261 | Adrenergic signaling in cardiomyocytes | 44 | 0.000132251 | Cacng8/Ppp2r2c/Adcy1/Camk2d/Camk2b/LOC100362453/Slc8a3/Calml4/Slc8a2/Scn7a/Cacnb4/Cacnb3/Camk2a/Cacnb1/Agtr2/Plcb4/Akt2/Pln/Ppp2r3c/Atp1a1/Cacna1f/Scn4b/Gnas/Ppp2r2a/Adcy8/Atf6b/Ppp1r1a/Cacna2d2/Adra1a/Cacna1c/Cacng5/Ppp2r2b/Atp2b3/Calm2/Agtr1a/Mapk14/Ppp2r3a/Adra1d/Adra1b/Adcy4/Mapk12/Prkacb/Ppp1cc/Cacna1d |
| rno04270 | Vascular smooth muscle contraction | 40 | 0.000181617 | Gna11/AABR07062799.2/Adcy1/Gucy1a1/Calml4/Ppp1r12c/Mrvi1/Ramp1/Gucy1b1/Acta2/Mylk/Rock1/Prkch/Nppc/Plcb4/Ednra/Edn3/Prkcd/Pla2g12a/Cacna1f/Gnas/Adora2a/Adcy8/Adra1a/Myh11/Cacna1c/Rhoa/Pla2g5/Calm2/Agtr1a/Adm/Map2k1/Adra1d/Prkce/Adra1b/Myl6/Adcy4/Prkacb/Ppp1cc/Cacna1d |
| rno04728 | Dopaminergic synapse | 41 | 0.000277219 | Ppp2r2c/Drd2/Gria4/Camk2d/Camk2b/Drd1/LOC100362453/Calml4/Gng4/Arrb2/Scn1a/Camk2a/Gng11/Gnb4/Plcb4/Akt2/Ppp2r3c/Gng5/Gria1/Gnas/Comt/Ppp2r2a/Gsk3a/Atf6b/Cacna1c/Ppp2r2b/Mapk9/Th/Gnal/Maob/Calm2/Drd3/Mapk14/Cacna1a/Ppp2r3a/Gngt1/Kcnj6/Mapk12/Prkacb/Ppp1cc/Cacna1d |
| rno04144 | Endocytosis | 71 | 0.000403225 | Psd/Hras/Ehd1/Wipf3/Capza1/Iqsec2/Sh3gl1/Agap2/Dnm3/Mvb12b/Arrb2/Pip5k1c/Pld1/Arf5/Smad3/Wwp1/Zfyve16/Clta/LOC688655/Sh3kbp1/-/Hspa1b/Agap3/Git1/AABR07033570.2/Ccr5/Snx5/Wipf2/Epn1/Arpc2/Arpc1a/Fkbp11/Usp8/AABR07011951.1/Cyth1/AABR07046136.1/Iqsec3/Vps37b/Cblc/Stambp/Ehd3/Src/Chmp6/Cxcr4/RT1-T24-4/RT1-M6-2/Cdc42/Cav2/Epn2/Snx6/Rhoa/RT1-CE7/Rab5a/Vps4b/AABR07048992.1/Rab11b/Grk1/Igf2r/RT1-M3-1/Chmp4bl1/Dab2/Capzb/Eea1/Tsg101/Arfgap3/Pip5kl1/Acap3/Rnf41/Cyth3/Hgs/RT1-A2 |
| rno04925 | Aldosterone synthesis and secretion | 30 | 0.000651163 | Gna11/AABR07062799.2/Adcy1/Camk2d/Camk2b/Calml4/Cacna1h/Dagla/Camk2a/Kcnk9/Plcb4/Kcnk3/Atp1a1/Cacna1f/Gnas/Adcy8/Atf6b/Cacna1c/Cacna1i/Nr4a1/Atp2b3/Calm2/Agtr1a/Prkd2/Orai1/Prkce/Adcy4/Pomc/Prkacb/Cacna1d |
| rno04024 | cAMP signaling pathway | 54 | 0.001534175 | Drd2/Gria4/Adcy1/Camk2d/Camk2b/Drd1/Pik3r2/Calml4/Sox9/Pld1/Grin3a/Rap1a/Vipr2/Camk2a/Pde4b/Chrm2/Rock1/Acox1/Tiam1/Akt2/Ednra/Pln/Hcn4/Jun/Edn3/Atp1a1/Gria1/Cacna1f/Vav2/Gnas/Adora2a/Adcy8/Cacna1c/Rhoa/Atp2b3/Mapk9/Calm2/Pak1/Gli3/Ghsr/Htr1f/Orai1/Map2k1/Grin1/Hcar2/Adcy4/Pomc/Htr1d/Chrm1/Prkacb/Ppp1cc/Cacna1d/Bad/Hcar1 |
| rno04260 | Cardiac muscle contraction | 27 | 0.002007283 | Cacng8/Cox8a/AABR07015346.1/Uqcr11/Slc8a3/Slc8a2/Uqcrb/Cacnb4/Cacnb3/Cox5b/Cacnb1/Cox7a2l2/Atp1a1/Cacna1f/Casq2/Asph/Cox6c/Cacna2d2/Mt-cyb/Cacna1c/Cox6a1/Cacng5/Mt-co1/Trdn/Mt-co2/Uqcrc2/Cacna1d |
| rno04010 | MAPK signaling pathway | 72 | 0.003297247 | Cacng8/Hras/Fgfr1/Gadd45b/Dusp6/Cacna1h/Tek/Arrb2/Crk/LOC688655/Max/Ngfr/Rap1a/Cacnb4/Cacnb3/Hspa1b/Dusp4/Dusp8/Hspb1/Cacnb1/Map3k5/Dusp5/AC239701.4/Akt2/Jun/AABR07011951.1/Pdgfb/Fgf22/Cacna1f/Mef2c/Erbb4/Elk1/Efna3/AABR07007642.1/Efna2/Cacna2d2/Mras/Ngf/Cdc42/Fgf2/Taok3/Traf2/Cacna1c/Mapk8ip2/Casp3/Cacna1i/Cacng5/AABR07048992.1/Rasgrp2/Srf/Nr4a1/Mapk9/Dusp7/Il1r1/-/Pak1/Fgf7/Ntf3/Mapk14/Tgfb3/Cacna1a/Rps6ka3/Map2k1/-/Mknk2/AABR07034750.1/Ptpn7/Mapk12/Map2k5/Jund/Prkacb/Cacna1d |
